# Genomic characterization of respiratory *Elizabethkingia*: antimicrobial resistance and strain relatedness

**DOI:** 10.3389/fmicb.2026.1815762

**Published:** 2026-04-10

**Authors:** Yongpeng Shang, Junyou Lin, Jie Sun, Zhijun Zhang, Shuying Yuan, Fangyou Yu, Guiqin Sun

**Affiliations:** 1 Department of Clinical Laboratory, Shanghai Pulmonary Hospital, School of Medicine, Tongji University, Shanghai, China; 2 School of Medical Technology and Information Engineering, Zhejiang Chinese Medical University, Hangzhou, China; 3 Department of Clinical Laboratory, The Afffliated Taian City Central Hospital of Qingdao University, Taian, China; 4 Department of Clinical Laboratory, Jiaxing Maternity and Child Health Care Hospital, Affiliated Women and Children Hospital of Jiaxing University, Jiaxing, China

**Keywords:** antimicrobial resistance, core-genome SNPs, *Elizabethkingia*, host immune response, severe respiratory infection, whole-genome sequencing

## Abstract

*Elizabethkingia* species are emerging multidrug-resistant opportunistic pathogens associated with high mortality in critically ill patients. However, the genomic epidemiology and mechanisms of fluoroquinolone resistance in respiratory *Elizabethkingia* isolates remain incompletely understood. The objective of this study was to characterize the clinical, genomic, and resistance features of respiratory *Elizabethkingia* isolates from a tertiary pulmonary hospital and to explore the structural basis of fluoroquinolone non-susceptibility associated with DNA gyrase subunit A (GyrA) S83I substitution. A total of 18 non-duplicate *Elizabethkingia* isolates from 16 patients with severe pulmonary disease at a tertiary hospital (2024–2025) were included. Whole-genome sequencing was performed for species identification, pangenome analysis, and core-genome SNP-based phylogenetic reconstruction. Antimicrobial susceptibility testing was conducted using the VITEK^®^ 2 system. Mutations in the quinolone resistance-determining region (QRDR) were analyzed, and the structural impact of the GyrA S83I substitution was evaluated by molecular docking. Among the 18 isolates, 15 were identified as *Elizabethkingia anophelis* and 3 as *Elizabethkingia meningoseptica*. Pangenome analysis demonstrated an open genome structure (γ = 0.156). Core-genome phylogeny revealed distinct clade-specific clustering, with seven highly related *E. anophelis* isolates in Clade B, suggesting potential nosocomial transmission. All isolates exhibited broad resistance to multiple antimicrobial classes, whereas minocycline retained full *in vitro* activity. Fluoroquinolone non-susceptibility was strongly associated with lineage and significantly correlated with the GyrA S83I substitution. Molecular docking analysis showed that this substitution reduced the binding affinity of ciprofloxacin and levofloxacin to GyrA (ΔΔG ≈ 3–6 kcal/mol). However, because GyrA S83I was largely lineage-restricted, its independent contribution could not be disentangled from clade background, and several non-susceptible isolates lacked S83I, indicating additional mechanisms, our integrated genomic and structural findings underscore the critical interplay between localized clonal expansion and target-site adaptation in driving fluoroquinolone resistance in clinical *Elizabethkingia* strains.

## Introduction

The genus *Elizabethkingia*, an emerging environmental pathogen, is widely distributed in diverse habitats, including soil, freshwater ecosystems, and the gastrointestinal tracts of insects and amphibian (Lei et al., 2019; Hem et al., 2022). To date, seven species have been identified within this genus: *E. anophelis*, *E. meningoseptica*, *Elizabethkingia miricola* (*E. miricola*), *Elizabethkingia bruuniana* (*E. bruuniana*), *Elizabethkingia ursingii* (*E. ursingii*), *Elizabethkingia occulta* (*E. occulta*), and *Elizabethkingia argenteiflava* (*E. argenteiflava*) (Takei et al., 2025). In recent years, these species have increasingly been recognized in clinical settings as rare but clinically significant opportunistic pathogens (Cai et al., 2025). Although the overall incidence of *Elizabethkingia* infections remains low, the associated in-hospital mortality rate is notably high, ranging from 20 to 60% (Lin et al., 2019b). Despite this clinical severity, the precise mechanisms underlying the pathogenicity of *Elizabethkingia* in human infections remain poorly understood.

Infections caused by *Elizabethkingia* species are predominantly bloodstream infections, although they have also been associated with pneumonia, purulent arthritis, infectious ascites, meningitis, and ocular infections (Figueroa Castro et al., 2017; Bulagonda et al., 2018; Lin et al., 2019b). These infections primarily affect critically ill individuals with compromised host defenses, such as patients with malignancies, organ transplant recipients, those with prolonged stays in the intensive care unit (ICU), and individuals with advanced pulmonary diseases, and are frequently linked to high mortality rates (Jean et al., 2014; Han et al., 2017). Moreover, *Elizabethkingia* demonstrates broad resistance to multiple classes of antibiotics, including β-lactams, aminoglycosides, and carbapenems, largely driven by its highly conserved chromosomal resistome—severely limiting therapeutic options for clinicians (Han et al., 2017).

Currently, research on *Elizabethkingia* has primarily focused on antibiotic susceptibility profiles and clinical case reports, whereas systematic genomic studies remain limited, particularly concerning respiratory isolates from Chinese patients with pulmonary diseases (Hu et al., 2022; Lee et al., 2023). To address this gap, the present study conducted a comprehensive clinical and genomic investigation of *Elizabethkingia* strains isolated from the respiratory tract of critically ill patients with pulmonary conditions in China. By integrating whole-genome sequencing, core genome single-nucleotide polymorphism (cgSNP)-based population structure analysis, antibiotic susceptibility testing, and characterization of resistance genes and mutational profiles, this study aims to elucidate the population structure of *Elizabethkingia* within nosocomial infection settings and enhance understanding of the genetic basis underlying antibiotic resistance.

## Materials and methods

### Study design and ethical approval

This retrospective observational study was conducted at Shanghai Pulmonary Hospital, a tertiary referral center specializing in respiratory diseases. The study protocol was reviewed and approved by the institutional ethics committee of Shanghai Pulmonary Hospital (Ethical approval no. K25–553). Given the retrospective nature of the study and the use of anonymized clinical data, informed consent was waived in accordance with institutional policies.

### Patient inclusion and isolate grouping

Medical records from January 2024 to June 2025 were screened. A total of 18 non-duplicate *Elizabethkingia* isolates recovered from 16 patients were included in the analysis. Two patients contributed paired isolates obtained from distinct anatomical sites. These isolates were retained as independent samples for genomic analyses, as whole-genome sequencing confirmed that each isolate represented a genetically distinguishable strain. For clinical and immunological analyses, patient-level data were used, with paired isolates linked to the same patient where appropriate.

### Microbiological identification and antimicrobial susceptibility testing

Clinical specimens, including bronchoalveolar lavage fluid (BALF), pleural effusion, sputum, urine, and catheter tips, were processed following routine microbiological procedures. Species identification was performed using matrix-assisted laser desorption/ionization time-of-flight mass spectrometry (MALDI-TOF MS; bioMérieux, France).

Antimicrobial susceptibility testing (AST) was performed using the VITEK^®^ 2 Compact system (bioMérieux, France). Minimum inhibitory concentrations (MICs) were interpreted according to the Clinical and Laboratory Standards Institute (CLSI) M100 guidelines, 35th edition, using the criteria for “other non-*Enterobacterales*” when available (Clinical and Laboratory Standards Institute [CLSI], 2025). As no species-specific CLSI breakpoint is available for tigecycline in *Elizabethkingia* spp., tigecycline MICs were interpreted using the FDA *Enterobacterales* criteria (susceptible, ≤ 2 μg/mL; intermediate, 4 μg/mL; resistant, ≥ 8 μg/mL)^1^ for descriptive purposes only (Kelesidis et al., 2008; Lin et al., 2019c). Therefore, AST results are presented primarily as MIC distributions, and categorical interpretations were applied cautiously in comparative analyses.

### Microbiological criteria

Respiratory isolation of *Elizabethkingia* in mechanically ventilated ICU patients may represent infection or colonization. Given the retrospective nature of the study and incomplete standardized radiographic/quantitative culture data, we did not attempt to definitively adjudicate infection versus colonization. Predominant growth and/or repeat isolation within 72 h were recorded as indicators of microbiological persistence to reduce the likelihood of incidental contamination.

### Clinical data collection

Demographic characteristics, underlying diseases, clinical course, antimicrobial exposure, and laboratory data were extracted from the hospital Laboratory Information System (LIS) and electronic medical records. For each patient, the most recent laboratory results obtained within 7 days of the first positive culture were selected to reflect the clinical and immunological status surrounding the initial isolation event while minimizing temporal heterogeneity introduced by subsequent treatment and disease progression. Laboratory parameters included complete blood counts, liver and renal function tests, coagulation indices, and inflammatory and immune markers, including cytokine profiling.

### Genome sequencing, assembly and bioinformatic analysis

After initial recovery from clinical specimens, the isolates were purified by a single subculture on blood agar plates. Pure bacterial growth was collected, suspended in 30% glycerol, and stored at -80°C until further analysis. Prior to whole-genome sequencing, the isolates were recovered from frozen stocks and streaked onto blood agar plates for 12 h of revival culture. A single pure colony was then inoculated into 200 mL of LB broth and cultured at 37°C for approximately 12 h with shaking at 150 rpm. *Elizabethkingia* strains were streaked on LB agar plates and incubated at 37°C for 12 h. A single colony was inoculated into 200 mL of LB broth and cultured at 37°C for approximately 12 h with shaking at 150 rpm. Bacterial cells were harvested by centrifugation at 12,000 × g for 10 min. Genomic DNA was extracted using a bacterial genomic DNA extraction kit (magnetic bead method; Majorbio, Shanghai, China) according to the manufacturer’s instructions. DNA concentration was measured using a Quantus Fluorometer with the PicoGreen dsDNA assay, and DNA integrity was evaluated by native agarose gel electrophoresis. High-quality DNA was defined as DNA with a main band ≥ 3 kb, a non-viscous solution, no pigment or suspended material, no severe contamination by RNA, proteins, or polysaccharides, and a total amount sufficient for one standard library construction.

Whole-genome sequencing was performed on the Illumina next-generation sequencing platform using paired-end reads. Raw reads were quality-filtered using fastp (version 0.19.6) (Chen et al., 2018) to remove adapter sequences and low-quality bases. Clean reads were assembled *de novo* using SOPA (version 2.04) (Luo et al., 2012). Genome completeness and contamination were evaluated using CheckM2 to ensure assembly quality (Chklovski et al., 2023). The assembled genomes have been deposited in the NCBI Sequence Read Archive (SRA) under BioProject accession number PRJNA1312355.

### Comparative genomic and phylogenetic analysis

Species identification was confirmed by average nucleotide identity (ANI) analysis using pyani, with pairwise genome comparisons performed using MUMmer (version 3.23) (Kurtz et al., 2004). ANI values ≥ 95% were considered indicative of species-level identity.

Genome annotation was performed using PGAP (version 1.2.1) (Richter and Rosselló-Móra, 2009). Based on annotated protein-coding genes, pangenome analysis was conducted using Roary (version 3.9.1) (Page et al., 2015) with default parameters to classify core, accessory, and strain-specific gene families. Pangenome openness was evaluated using Heaps’ law modeling (Tettelin et al., 2008).

CgSNPs were identified using a reference-based mapping approach. For *E. anophelis*, strain GCA_002023665.2 was used as the reference genome, whereas for *E. meningoseptica*, strain GCA_900475375.1 served as the reference. SNPs were identified relative to the species-specific reference genome, and concatenated core-SNP alignments were generated after filtering low-quality positions.

To place the study isolates in a broader phylogenetic framework, additional publicly available reference genomes were retrieved from the NCBI Assembly database, including *E. miricola* EM_CHUV (GCA_001483145.1), *E. ursingii* G4123 (GCA_002022125.1), *E. occulta* G4070 (GCA_002023715.1), and *E. bruuniana* G0146 (GCA_002024805.1). *Chryseobacterium indologenes* NCTC10796 (GCA_900460995.1) was included as the outgroup.

Maximum-likelihood phylogenetic trees were reconstructed using IQ-TREE (version 3.0.1)(Nguyen et al., 2015). The best-fit nucleotide substitution model was selected automatically using ModelFinder implemented in IQ-TREE. Branch support was assessed using 1,000 ultrafast bootstrap replicates.

### Antimicrobial resistance determinant analysis

Antimicrobial resistance determinants were identified by BLASTP (version 2.3.0) searches against the Comprehensive Antibiotic Resistance Database (CARD, version 1.1.3) (Jia et al., 2017). Antimicrobial resistance determinants were identified using the Comprehensive Antibiotic Resistance Database (CARD). CARD-annotated loci may include both intrinsic/chromosomally encoded resistance determinants and putative acquired resistance genes. Resistance-associated mutations in the QRDR of GyrA were manually examined, with particular emphasis on fluoroquinolone resistance–associated substitutions.

### Molecular docking analysis

Molecular docking of GyrA with the fluoroquinolone antibiotics ciprofloxacin and levofloxacin was performed to investigate the structural basis of mutation-associated antibiotic resistance. The three-dimensional structures of both wild-type and mutant GyrA were predicted using AlphaFold3 via the AlphaFold Server (Niemyska et al., 2022). The Ser83Ile substitution was introduced into the wild-type protein structure to generate the mutant model for comparative analysis.

Semi-flexible molecular docking between the GyrA active site and the ligands (ciprofloxacin and levofloxacin) was conducted using UCSF DOCK 6.9 (Mukherjee et al., 2010). Binding conformations were evaluated based on grid scores, and binding energies (kcal/mol) were used to identify the most energetically favorable poses. Protein–ligand interactions were visualized and analyzed using PyMOL (Osipovitch et al., 2015).

### Statistical analysis

Statistical analyses were performed using SPSS version 27.0. Continuous variables were summarized as means ± standard deviation or medians with interquartile ranges, as appropriate. Categorical variables were presented as counts and percentages. Group comparisons were conducted using parametric or non-parametric tests according to data distribution.

## Results

### Clinical characteristics of *Elizabethkingia* species infections

Between 2024 and 2025, a total of 18 *Elizabethkingia* isolates were obtained from clinical specimens collected from 16 patients at Shanghai Pulmonary Hospital (Table 1). The median age was 69.7 years, and 62.5% were male. The most common specimen sources were BALF (*n* = 8) and sputum (*n* = 5), followed by hydrothorax (*n* = 2) and urinary catheter tip (*n* = 1) (Table 1). All patients were admitted to the ICU and required invasive mechanical ventilation during their hospital course. The most frequent underlying conditions included lung transplantation, malignant lung tumors, and interstitial lung disease. Overall, six patients (37.5%) died during hospitalization. Empirical regimens were heterogeneous and frequently included combination therapy. Minocycline was the most commonly used agent (9/16, 56.3%), followed by doxycycline and levofloxacin.

**TABLE 1:** Clinical characteristics, treatment strategies, and outcomes of patients with *Elizabethkingia* species infection.

Patient ID	Age	Sex	Underlying condition	Specimen types	Empirical antimicrobial regimen	Treatment outcome
1	70	Male	Right lung adenocarcinoma	Hydrothorax	Vancomycin + Tigecycline + Levofloxacin	Survival
2	75	Male	Interstitial lung disease	Sputum	Levofloxacin + Minocycline + Cefoperazone-sulbactam	Death
3	68	Male	Lung transplantation	Sputum	Minocycline + Ceftazidime-avibactam	Survival
4	61	Female	Left lower lung surgery	BALF	Levofloxacin + Doxycycline	Survival
5	84	Female	Severe pneumonia	Sputum	Doxycycline + Ceftazidime-avibactam	Survival
6	77	Male	Lung transplantation	BALF	Vancomycin + Minocycline	Death
7	79	Male	Bronchiectasia	BALF	Minocycline	Death
8	83	Female	Encapsulated pleural effusion	Hydrothorax	Tigecycline	Survival
9	68	Female	Interstitial lung disease	BALF	Tigecycline + Ceftazidime-avibactam	Death
10	75	Male	Malignant lung tumor	BALF	ceftazidime-avibactam + Vancomycin	Death
11	69	Male	Squamous cell carcinoma of the right lung	BALF	Doxycycline + Ceftazidime-avibactam	Survival
12	65	Female	Lung transplantation	Indwelling urinary	Doxycycline + Sulfamethoxazole	Survival
13	65	Male	Lung transplantation	BALF	Minocycline	Survival
14	66	Male	Lung transplantation	Sputum	Cefoperazone-sulbactam + Levofloxacin + Minocycline	Death
15	60	Female	Severe pneumonia	Sputum	Doxycycline + Sulfamethoxazole	Survival
16	50	Male	Lung transplantation	BALF	Minocycline + Eracycline	Survival

BALF, Bronchoalveolar Lavage Fluid.

Laboratory analyses demonstrated systemic inflammation in patients with severe pulmonary disease from whom *Elizabethkingia* was isolated. These findings should be interpreted as descriptive host-context data rather than *Elizabethkingia*-specific immune signatures, given the retrospective design and the presence of substantial clinical confounding. Median hs-CRP and PCT levels were 48.50 mg/L (IQR 17.10–73.70) and 1.22 ng/mL (IQR 0.16–3.56), respectively, both exceeding normal ranges (Table 1). Proinflammatory cytokines were markedly elevated, including IL-6 (median 42.05 pg/mL) and IL-8 (mean 167.53 pg/mL), while the anti-inflammatory cytokine IL-10 (median 11.54 pg/mL) was also increased, suggesting complex immune activation (Table 2). Hematologic abnormalities included anemia (mean hemoglobin 88 g/L), lymphopenia, and neutrophilia, accompanied by hypoalbuminemia and reduced total protein (Table 2). Coagulation parameters indicated hypercoagulability, with elevated D-dimer and FDP and reduced AT-III (Table 2).

**TABLE 2:** Summary of hematological, biochemical, and immunological parameters in 16 patients infected with *Elizabethkingia* species.

Category	Variable	Overall, *N* = 16	Reference range	Interpretation
Inflammatory markers	**hs-CRP (mg/L)**	**48.50 (17.10, 73.70)**	0–3	↑↑↑
	**PCT (ng/mL)**	**1.22 (0.16, 3.56)**	0.00–0.50	↑↑
	IL-4	3.08 (2.71)	0.00–3.00	↑
	**IL-6**	**42.05 (18.74, 273.14)**	0.00–5.30	↑↑↑
	**IL-8**	**167.53 (147.08)**	0.00–20.6	↑↑↑
	**IL-10**	**11.54 (7.91, 24.62)**	0.00–4.91	↑↑
	TNF	2.02 (1.40, 3.32)	0.00–4.60	—
	IFN-γ	3.01 (1.41, 4.56)	0.00–7.42	—
Liver and renal Function markers	ALT (U/L)	23.85 (14.73, 49.95)	10–49	—
	AST (U/L)	25.85 (17.10, 30.40)	0–34	—
	**TP (g/L)**	**53.91 (6.56)**	66.0–83.0	↓
	**ALB (g/L)**	**29.85 (26.35, 33.38)**	35.0–52.0	↓
	GLB (g/L)	22.89 (4.90)	20–45	—
	**UA (μ mol/L)**	**161.53 (70.57)**	M: 220–547; F: 184–464	↓
	**BUN (mmol/L)**	**10.23 (4.75)**	2.8–7.2	↑
	Cr (μmol/L)	46.75 (41.25, 100.83)	M: 53–97; F: 44–71	—
Hematologic markers	**RBC counts (× 10^12^/L)**	**2.83 (0.79)**	M: 4.3–5.8; F: 3.8–5.1	↓
	**Hb (g/L)**	**88 (23.63)**	M: 130–175; F: 115–150	↓
	WBC counts (× 10^9^/L)	9.88 (5.94)	3.5–9.5	↑
	NEUT (× 10^9^/L)	7.75 (4.48, 12.11)	2.00–7.70	↑
	LYMPH (× 10^9^/L)	0.62 (0.31)	0.80–4.00	↓
	PLT (× 10^9^/L)	189.25 (84.98)	125–350	—
Coagulation markers	PT (s)	12.10 (11.25, 12.68)	11–13	—
	INR	1.08 (1.01, 1.13)	1.0–1.1	—
	**APTT (s)**	**29.00 (27.43, 31.00)**	31–43	↓
	TT (s)	15.26 (1.69)	16–18	↓
	FIB (g/L)	3.43 (1.07)	2–4	—
	**D-Dimer (mg/L)**	**2.05 (1.37, 3.80)**	negative	↑
	FDP (mg/L)	6.20 (3.61, 7.82)	0–5	↑
	AT-III (%)	67.50 (21.64)	80–120	↓

hs-CRP, high-sensitivity C-reactive protein; PCT, procalcitonin; IL-4, interleukin-4; IL-6, interleukin-6; IL-8, interleukin-8; IL-10, interleukin-10; TNF, tumor necrosis factor; IFN-γ, interferon-gamma; ALT, alanine aminotransferase; AST, aspartate aminotransferase; TP, total protein; ALB, albumin; GLB, globulin; UA, uric acid; BUN, blood urea nitrogen; Cr, creatinine; RBC, red blood cell; Hb, hemoglobin; WBC, white blood cell; NEUT, neutrophil count; LYMPH, lymphocyte count; PLT, platelet count; PT, prothrombin time; INR, international normalized ratio; APTT, activated partial thromboplastin time; TT, thrombin time; FIB, fibrinogen; D-dimer, D-dimer; FDP, fibrin/fibrinogen degradation products; AT-III, antithrombin III.↑ above reference range; ↓ below reference range; — within reference range; bold font: significant difference.

### Clinical antimicrobial resistance characteristics of *Elizabethkingia* isolates

All clinical isolates of *Elizabethkingia* spp. exhibited uniform resistance to piperacillin–tazobactam, ceftazidime, cefepime, imipenem, meropenem, amikacin, tobramycin, and polymyxin B. Intermediate resistance was observed for levofloxacin (11/18, 61.11%), ciprofloxacin (12/18, 66.67%), and tigecycline (7/18, 41.18%), whereas trimethoprim–sulfamethoxazole (4/18, 23.53%) demonstrated the lowest resistance rate among the tested agents. Notably, minocycline retained full *in vitro* activity against all isolates (Table 3).

**TABLE 3:** Antimicrobial susceptibility patterns of *Elizabethkingia* species Isolates.

Variable	Susceptible	Intermediate	Resistant
Piperacillin-tazobactam	0	0	18 (100)
Cefoperazone-sulbactam	3 (16.67)	6 (33.33)	9 (50)
Ceftazidime	0	0	18 (100)
Cefepime	0	2 (11.11)	16 (88.89)
Imipenem	0	0	18 (100)
Meropenem	0	0	18 (100)
Amikacin	0	0	18 (100)
Tobramycin	0	0	18 (100)
Ciprofloxacin	5 (27.79)	1 (5.56)	12 (66.67)
Levofloxacin	7 (38.89)	0	11 (61.11)
Doxycycline	12 (70.59)	2 (11.76)	3 (17.65)
Minocycline	17 (100)	0	0
Tigecycline	4 (23.53)	6 (35.29)	7 (41.18)
Colistin	0	0	18 (100)
Trimethoprim-sulfamethoxazole	13 (76.47)	0	4 (23.53)

Doxycycline, doxycycline; Minocycline, minocycline; Tigecycline, tigecycline; Co-trimoxazole, trimethoprim-sulfamethoxazole. Susceptibility results for Doxycycline, Minocycline, Tigecycline, and Co-trimoxazole were available for 17 isolates only, because isolate FK-2 was not tested for these four agents.

### Genomic and general features of *Elizabethkingia* strains

To further investigate the genetic basis of the resistance phenotypes, we performed whole-genome sequencing on all 18 *Elizabethkingia* isolates using Illumina short-read technology. The assembled genomes ranged from 3.88 to 4.09 Mb (mean: 4.02 Mb) and had a mean GC content of 35.72%. They contained 3,503 to 3,788 predicted protein-coding sequences (CDSs) per genome, with an average of 3,693 CDSs (Supplementary Table 1). Genome quality assessment using CheckM2 revealed high completeness (≥ 99.7%; mean: 100%) and low contamination levels (≤ 0.28%; mean: 0.18%), indicating that all assemblies fulfilled the recommended criteria for downstream comparative genomic analyses (Supplementary Table 1).

**FIGURE 1:**
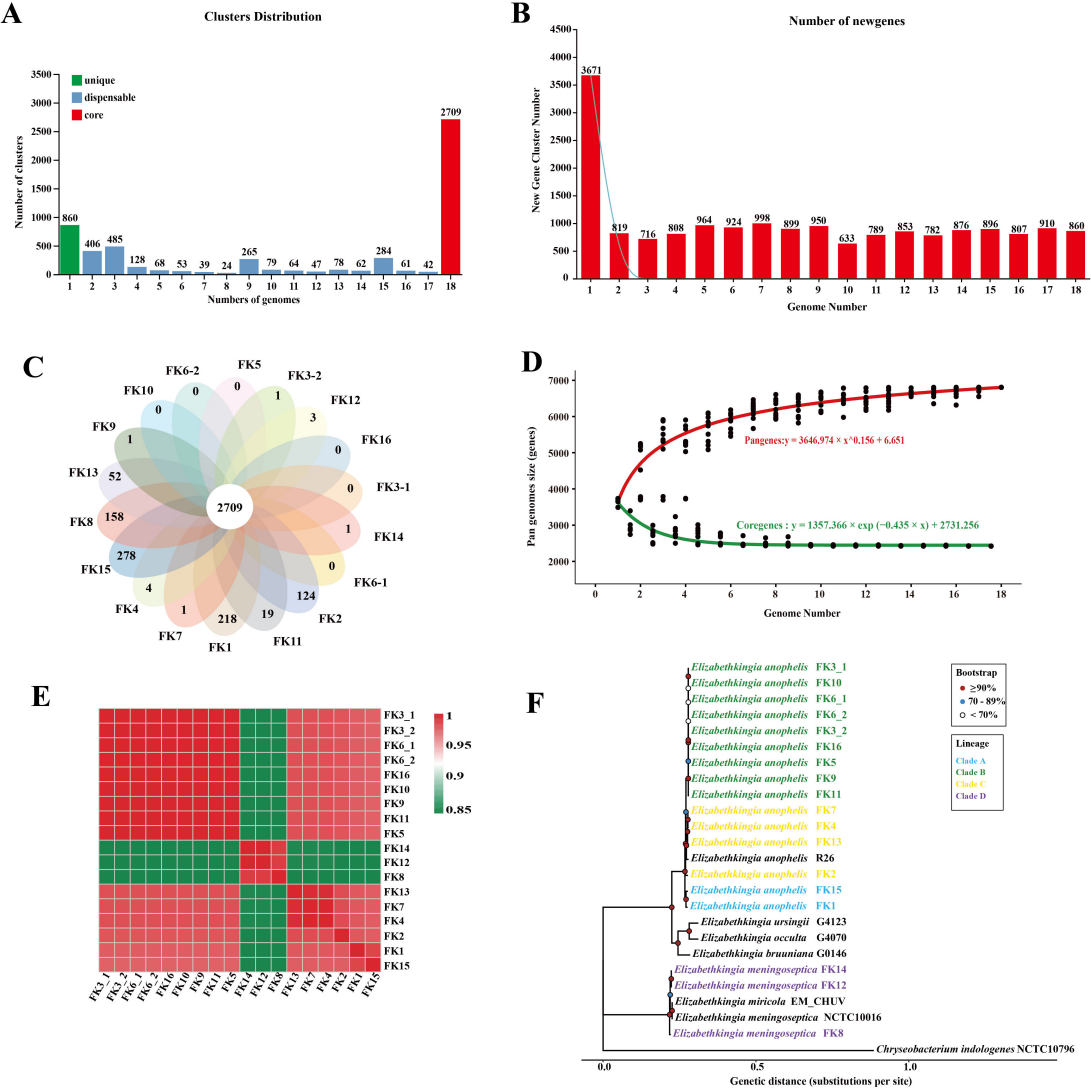
Pan-genome and core-genome analysis of *Elizabethkingia* strains. **(A)** Distribution frequency of gene clusters across the 18 sequenced genomes. **(B)** Number of novel gene clusters added with each additional genome. **(C)** Flower plot depicting the core genome size at the center and the strain-specific gene clusters in the petals. **(D)** Cumulative curves illustrating the decreasing trend of core gene clusters (green) and the increasing trend of pan gene clusters (red) as genome sampling increases. **(E)** ANI heatmap of *Elizabethkingia* Species. **(F)** Maximum-likelihood phylogenetic tree based on core-genome SNPs of the 18 *Elizabethkingia* clinical isolates and representative public reference genomes. The tree was rooted using *C. indologenes* NCTC10796 as an outgroup. Node symbols indicate bootstrap support values based on 1,000 replicates. The scale bar represents genetic distance (substitutions per site).

The pan-genome analysis identified a total of 5,754 gene clusters, of which 2,709 were classified as core genes, and 696–1,016 were accessory genes (Figures 1A,B). Additionally, each strain harbors between 1 and 278 unique genes, with strain FK15 possessing the highest number of strain-specific genes (Figure 1C). The pan-genome curve was fitted using power-law regression based on Heap’s law. For N (number of genomes) = 18, the resulting model is described by the equation: y = 3646.974 × x^0.156 + 6.651, where y represents the total number of pan-genes and x denotes the number of genomes analyzed, with a power-law coefficient (γ) of 0.156. Since γ < 1, this indicates an open pan-genome, reflecting the ongoing acquisition of accessory genes with the addition of each new genome (Figure 1D). Core-genome analysis revealed a conserved set of 2,709 genes, with the number of shared genes decreasing as more genomes were incorporated. The core-genome trend approached saturation, as demonstrated by least-squares fitting of an exponential decay regression model to the mean values: y = 1357.366 × exp (-0.435 × x) + 2731.256. This result indicates that with increasing genome sampling, the core genome gradually stabilizes and the rate of shared gene loss progressively declines (Figure 1D).

Species identification and genomic relatedness were assessed using whole-genome average nucleotide identity (ANI) analysis. The majority of isolates exhibited extremely high pairwise ANI values (> 99.6%), indicating a high degree of genomic similarity. In contrast, isolates FK-8, FK-12, and FK-14 displayed markedly lower ANI values (approximately 84–85%) compared to the remaining strains, forming a distinct clade in the ANI-based clustering analysis, which suggests substantial genomic divergence from other *Elizabethkingia* isolates (Figure 1E). The phylogenetic tree constructed based on the core genome also confirmed that FK-8, FK-12, and FK-14 form a distinct clade (Figure 1F).

To further quantify within- and between-lineage relatedness, we calculated pairwise core-genome SNP distances. The nine isolates assigned to Clade B showed limited within-clade divergence (median, 4 SNPs; IQR, 13; range, 0–21; Supplementary Table 2), with many comparisons falling within a narrow SNP range. Specifically, seven isolates (FK3_1, FK3_2, FK5, FK6_1, FK6_2, FK10, and FK16) differed by ≤ 4 SNPs, and multiple isolate pairs were identical (0 SNPs), indicating near-indistinguishable genomic profiles. Two isolates (FK9 and FK11) exhibited relatively higher distances (12–21 SNPs) to the core cluster, suggesting early divergence while remaining within the same clade (Supplementary Table 3). In contrast, SNP distances between Clade B and other clades were orders of magnitude higher (Supplementary Table 2), supporting clear lineage separation. Together, these results indicate that Clade B constitutes a highly genetically cohesive cluster in our dataset.

### Resistome analysis of *Elizabethkingia*

**FIGURE 2:**
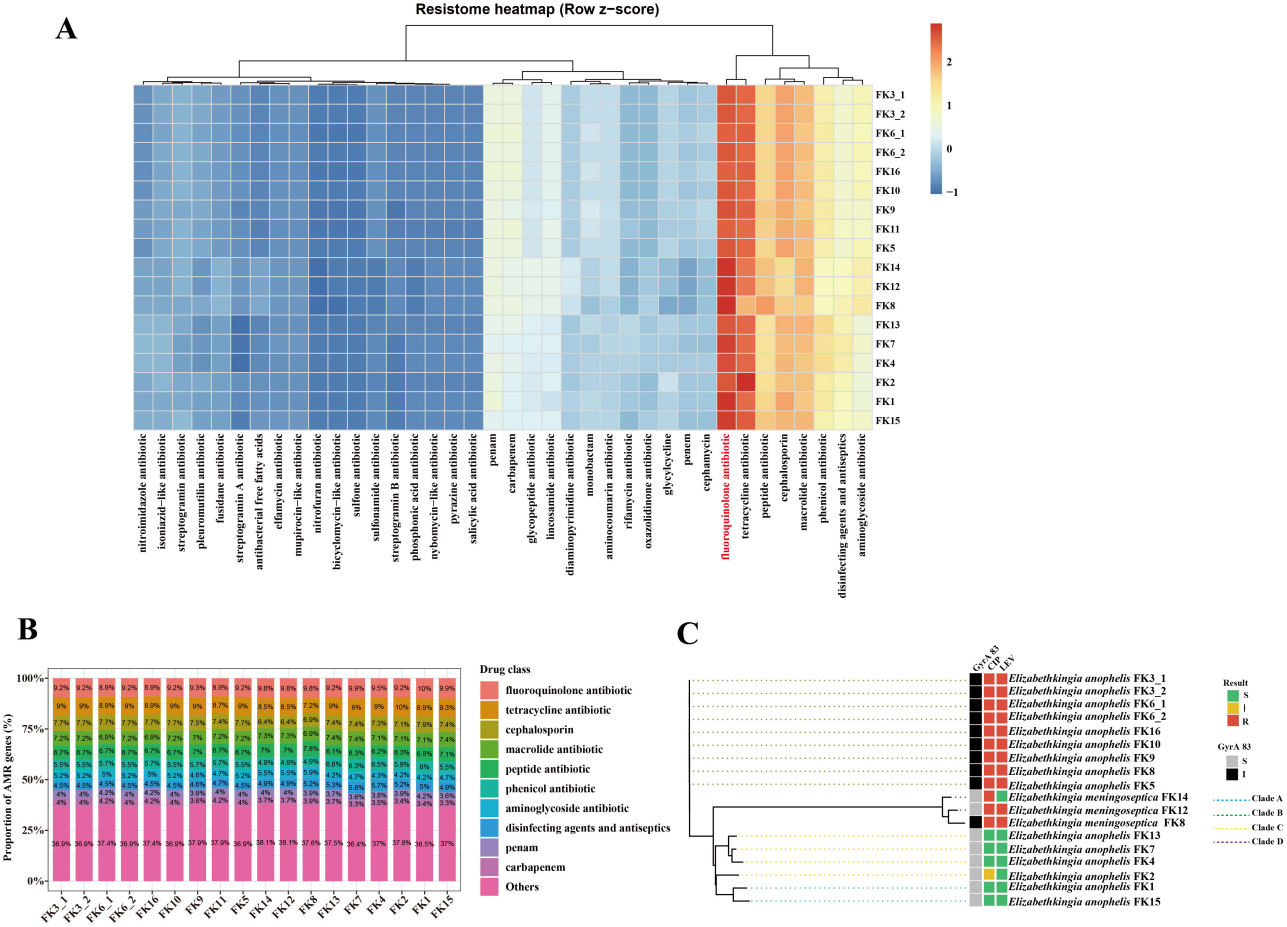
Resistome landscape and fluoroquinolone-associated features of 18 isolates. **(A)** Row z-score heatmap showing the relative enrichment of CARD-annotated resistance determinants across drug classes, **(B)** Proportional composition of CARD-annotated resistance determinants across major drug classes, **(C)** Phylogenetic distribution of the substitution at GyrA residue 83 and susceptibility grouping to ciprofloxacin (CIP) and levofloxacin (LEV).

We further performed resistome analysis on the 18 *Elizabethkingia* isolates and identified multiple CARD-annotated resistance determinants, which may represent a combination of intrinsic/chromosomally encoded resistance determinants and putative acquired resistance genes; therefore, these loci should not be interpreted exclusively as acquired resistance genes. The row z-score heatmap based on counts of CARD-annotated resistance determinants across drug classes indicated that the *Elizabethkingia* isolates shared broadly similar resistome composition patterns, while showing relative enrichment or depletion in certain drug classes (Figure 2A). Notably, fluoroquinolone-associated resistance determinants exhibited relatively higher enrichment in multiple isolates, suggesting that fluoroquinolone resistance may represent one of the major resistance features among these 18 *Elizabethkingia* strains. By integrating phenotypic AST data with our genomic analysis, we could delineate these resistance profiles into inherent and acquired categories. The uniform phenotypic resistance to β-lactams (including carbapenems), aminoglycosides, and polymyxin B across all isolates (Table 3) aligns with the highly conserved baseline resistome (Figure 2A). These widely shared determinants represent the inherent, chromosomally encoded resistance background characteristic of *Elizabethkingia* species. Conversely, variations in susceptibility to agents such as fluoroquinolones were primarily driven by acquired adaptive mechanisms.

To further visualize the proportional composition of CARD-annotated resistance determinants, a stacked bar plot of the Top10 drug classes plus Others revealed that the overall resistome composition was largely comparable among *Elizabethkingia* strains, with fluoroquinolone, tetracycline, cephalosporin, and macrolide resistance contributing substantially across isolates (Figure 2B). Although the overall proportions of drug classes differed only slightly, variations in fluoroquinolone-associated resistance were still observed among strains, indicating that differences in fluoroquinolone resistance phenotypes may not be fully explained solely by the number of CARD-annotated resistance determinants. Given the prominent multidrug-resistant phenotypes of the isolates, we specifically investigated the distribution of multidrug efflux pumps within the resistome. Annotation against the CARD database revealed that all 18 *Elizabethkingia* clinical isolates harbor an extensive and highly conserved repertoire of intrinsic efflux systems (Supplementary Table 8). The predicted efflux determinants span four major transporter families, prominently including the resistance-nodulation-cell division (RND) family, which is a key driver of MDR in Gram-negative bacteria. The identified RND pumps include multiple components of the *Mex* (*MexF*, *MexH*), *Ade* (*adeG*, *adeK*, *adeB*), *Cme* (*cmeB*), *Mdt*, and *Mux* efflux complexes. Furthermore, the ATP-binding cassette (ABC) transporter family (e.g., highly amplified *macB* with multiple copies per genome, *msbA*, *bcrA*), the major facilitator superfamily (MFS) (e.g., *emrB*, *emrY*, *lmrD*, *LmrS*), and the small multidrug resistance (SMR) family (e.g., *abeS*, *ykkC*, *ykkD*) were universally present. The ubiquitous presence of these diverse, broad-spectrum efflux systems likely provides a robust basal level of intrinsic resistance by synergistically extruding multiple antimicrobial classes, including fluoroquinolones, macrolides, and tetracyclines.

To elucidate the potential genetic basis underlying fluoroquinolone resistance in *Elizabethkingia*, we further integrated phylogenetic relationships with the mutation status of the fluoroquinolone target protein GyrA at residue 83 and susceptibility grouping for ciprofloxacin and levofloxacin (Figure 2C and Supplementary Table 4). The results showed that fluoroquinolone non-susceptible *Elizabethkingia* isolates exhibited lineage-associated clustering on the phylogenetic tree and were strongly concordant with the amino acid substitution at GyrA residue 83. Notably, substitution at GyrA residue 83 substitution was not restricted to a single *E. anophelis* clade in our collection; it was also observed in the *E. meningoseptica* isolate FK8, which was classified as fluoroquinolone non-susceptible. These findings indicate that fluoroquinolone non-susceptibility in our isolates is more directly supported by the target-site mutation at GyrA residue 83 than by the presence of a specific acquired resistance gene alone. At the same time, the observed co-occurrence between mutation status and phylogenetic background supports a lineage-associated pattern of fluoroquinolone non-susceptibility. Given the tight linkage between mutation status and phylogenetic background in this dataset, the independent effect of the substitution at GyrA residue 83 cannot be inferred from association alone.

In addition to *gyrA*, we systematically screened for sequence variations in topoisomerase IV (*parC* and *parE*), a known secondary target for fluoroquinolones (Supplementary Tables 5, 6). Several amino acid substitutions were identified among the isolates, including ParC T375A and S429A (highly prevalent in *E. anophelis* strains), as well as ParE K234R (in *E. meningoseptica* strains FK12 and FK14). However, sequence analyses revealed that all these identified substitutions are located distinctly outside the classical QRDRs of topoisomerase IV. The distribution of these distal mutations correlated strongly with phylogenetic lineages rather than specific fluoroquinolone resistance phenotypes, suggesting they represent naturally occurring genetic polymorphisms rather than primary resistance determinants, which further underscores the predominant role of the GyrA S83I substitution.

### Molecular interactions of levofloxacin and ciprofloxacin with *Elizabethkingia* spp.

**FIGURE 3:**
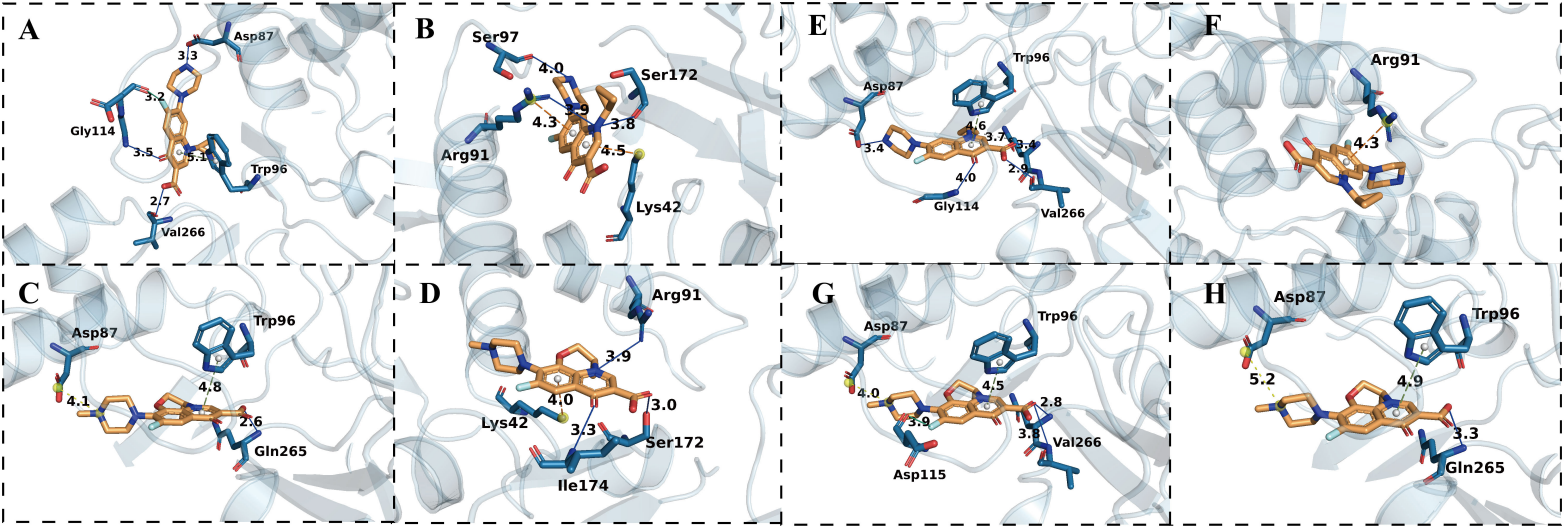
Molecular docking analysis of ciprofloxacin and levofloxacin with WT and S83I mutant GyrA from *E. anophelis* and *E. meningoseptica*. Ciprofloxacin binding is shown for *E. anophelis* WT **(A)** and S83I mutant **(B)**, and for *E. meningoseptica* WT **(E)** and S83I mutant **(F)**. Levofloxacin binding is shown for *E. anophelis* WT **(C)** and S83I mutant **(D)**, and for *E. meningoseptica* WT **(G)** and S83I mutant **(H)**. Blue solid lines indicate hydrogen bonds; green solid lines represent halogen bonds; green dashed lines denote π–π interactions; orange dashed lines indicate π–carbon interactions; and yellow dashed lines represent salt bridges.

Given that mutations in the target gene *gyrA* can modulate the binding affinity of fluoroquinolones, molecular docking was employed to quantitatively assess how such mutations affect ligand–protein interactions. Specifically, we identified the most energetically favorable ligand–protein complexes for ciprofloxacin and levofloxacin bound to both wild-type and mutant GyrA proteins, enabling a comparative analysis of binding modes and interaction profiles at the active site. More negative (i.e., more favorable) binding energies indicate stronger stabilization of the ligand–protein complex. The representative docking poses are shown in Figure 3, and the corresponding binding energy values are summarized in Table 4.

**TABLE 4:** Docking energy comparison between wild-type and S83I mutant GyrA.

Species	GyrA variant	Antibiotic	Docking energy (kcal/mol)
*E. anophelis*	WT	Ciprofloxacin	–37.939972
*E. anophelis*	S83I mutant	Ciprofloxacin	–31.999624
*E. anophelis*	WT	Levofloxacin	–36.863213
*E. anophelis*	S83I mutant	Levofloxacin	–34.241341
*E. meningoseptica*	WT	Ciprofloxacin	–41.090382
*E. meningoseptica*	S83I mutant	Ciprofloxacin	–35.086887
*E. meningoseptica*	WT	Levofloxacin	–37.882729
*E. meningoseptica*	S83I mutant	Levofloxacin	–33.924538

Molecular docking analysis revealed consistent structural perturbations in the QRDR of GyrA upon the S83I substitution, observed across both *E. anophelis* and *E. meningoseptica* (Figure 3). In WT complexes (Figures 3A,C,E,G), ciprofloxacin and levofloxacin adopted stable binding poses within the QRDR, engaging in multiple short-range interactions—including hydrogen bonds and hydrophobic contacts—with key residues Asp87, Arg91, Trp96, Gly114, and Val266. Interatomic distances for these interactions predominantly fell within canonical ranges for non-covalent ligand–protein stabilization (2.6–3.5 Å), indicative of well-ordered and energetically favorable binding interfaces.

In contrast, the S83I mutation induced measurable repositioning of both antibiotics within the binding pocket (Figures 3B,D,F,H). This was accompanied by a systematic increase in interatomic distances-commonly extending to 3.8–5.2 Å-and diminished coordination with functionally critical QRDR residues, most notably Asp87 and Arg91. These structural changes were consistently reflected in less favorable (i.e., less negative) docking scores across all comparisons (Table 4). Specifically, in *E. anophelis*, the predicted binding energy for ciprofloxacin decreased from -37.94 kcal/mol (WT) to -32.00 kcal/mol (S83I), and for levofloxacin from -36.86 to -34.24 kcal/mol. Analogous trends were observed in *E. meningoseptica*: ciprofloxacin binding energy shifted from -41.09 to -35.09 kcal/mol, and levofloxacin from -37.88 to -33.92 kcal/mol.

Collectively, the S83I substitution conferred a consistent reduction in predicted binding affinity for both fluoroquinolones, with ciprofloxacin exhibiting greater sensitivity to the mutation than levofloxacin-suggesting a structural basis for the differential impact on antibacterial activity.

## Discussion

Although *Elizabethkingia* species account for a small proportion of clinical isolates, our data demonstrate their capacity for localized clonal expansion and resistance convergence within a tertiary-care hospital. Recent epidemiological data indicate a progressive rise in healthcare-associated infections attributable to this genus, particularly among critically ill patients, where infections may progress to sepsis, meningitis, or pneumonia with substantial mortality risk (Teo et al., 2013; Lau et al., 2016; Figueroa Castro et al., 2017; Janda and Lopez, 2017).

Whole-genome sequencing defined the population structure of *Elizabethkingia* isolates from Shanghai Pulmonary Hospital and identified a genetically cohesive *E. anophelis* cluster (Clade B). Core-genome SNP analysis revealed minimal diversity (0–4 SNPs in most pairwise comparisons), consistent with recent common ancestry and genomic clustering (Supplementary Table 3). Although these findings are compatible with healthcare-associated dissemination or persistence from a shared reservoir, transmission routes cannot be inferred from genomic data alone (Teo et al., 2013; Figueroa Castro et al., 2017).

A limitation of this study is the inability to formally distinguish infection from colonization in mechanically ventilated ICU patients. Moreover, because all patients were critically ill and many had complex pulmonary disease and intensive care exposures, the observed inflammatory and immunological abnormalities cannot be directly attributed to *Elizabethkingia* alone. Therefore, our conclusions are confined to microbiological and genomic observations—namely, lineage-enriched fluoroquinolone non-susceptibility and tightly clustered genomes indicative of recent clonal expansion.

Given the high genomic homogeneity of this clonal cluster and its consistent antimicrobial resistance profile, our findings point to a close association between lineage-specific genetic background and the co-occurrence of key resistance determinants. Building on this observation, we prioritized fluoroquinolones—agents frequently used in the clinical management of *Elizabethkingia* infections yet exhibiting substantial inter-strain variability in susceptibility—for systematic investigation of their molecular resistance mechanisms (Lin et al., 2018; Jian et al., 2019; Lin et al., 2019a; Wang et al., 2020; Lin et al., 2022).

While intrinsic mechanisms confer universal baseline resistance to β-lactams and aminoglycosides, fluoroquinolone resistance in *Elizabethkingia* is predominantly mediated by amino acid substitutions within the QRDRs of DNA gyrase and topoisomerase IV (Lin et al., 2022). In this study, fluoroquinolone-resistant *E. anophelis* isolates predominantly harbored the GyrA S83I substitution, which was markedly enriched within Clade B. Notably, this mutation was not restricted to this lineage: the same GyrA S83I substitution was identified in a fluoroquinolone-resistant *E. meningoseptica* isolate (strain FK8) associated with meningitis and bacteremia. The occurrence of an identical QRDR substitution in phylogenetically distinct backgrounds supports the interpretation that GyrA S83I represents a recurrent adaptive mutation at a conserved resistance hotspot, rather than merely a clade-specific phylogenetic marker, consistent with parallel evolution under fluoroquinolone selection pressure.

Although GyrA S83I is strongly associated with the dominant *E. anophelis* clonal cluster in our cohort, its occurrence in *E. meningoseptica* indicates that position 83 constitutes a conserved mutational hotspot under fluoroquinolone selection pressure across the genus. This is firmly supported by multiple previous molecular epidemiological studies demonstrating that amino acid substitutions at Ser83 (e.g., S83I, S83R, or S83V) in GyrA are the most prevalent primary mechanisms conferring high-level fluoroquinolone resistance in clinical *Elizabethkingia* isolates (Lin et al., 2018; Jian et al., 2019; Lin et al., 2022). This pattern is consistent with parallel or convergent evolution, hereby structurally constrained residues repeatedly acquire similar substitutions to reduce drug binding affinity.

Although molecular docking studies in *Elizabethkingia* remain limited, they have been applied to target characterization in this genus, while previous studies on fluoroquinolone resistance in *Elizabethkingia* and related structural analyses in other bacterial systems have provided a useful framework for mechanistic interpretation (Madurga et al., 2008; Jian et al., 2019; Zhang et al., 2023). Building on these studies, we performed molecular docking simulations to further elucidate the structural basis of fluoroquinolone resistance associated with the GyrA S83I substitution. To further elucidate the structural basis of this genotype–phenotype association, molecular docking simulations were performed. The S83I substitution consistently increased the predicted binding energies (i.e., reduced binding affinity) of both ciprofloxacin and levofloxacin in GyrA orthologs from *E. anophelis* and *E. meningoseptica*, providing structural support for the observed fluoroquinolone resistance phenotype. Structurally, replacement of the polar Ser83 with hydrophobic isoleucine is predicted to alter the local hydrogen-bonding network within the QRDR microenvironment, particularly affecting interactions with Asp87 and Arg91, thereby destabilizing the drug–GyrA complex (Salehi et al., 2019; Liang et al., 2023). Notably, the energetic penalty associated with this substitution was greater for ciprofloxacin (ΔΔG ≈ 6.0 kcal/mol) than for levofloxacin (ΔΔG ≈ 3–4 kcal/mol), suggesting stronger dependence of ciprofloxacin on the polar microenvironment conferred by Ser83.

Collectively, these structural insights provide a mechanistic explanation for the enrichment of fluoroquinolone resistance within Clade B, while also supporting a broader role for GyrA S83I as a recurrent resistance-associated mutation across distinct *Elizabethkingia* lineages. However, because GyrA S83I remains enriched within a genetically cohesive cluster, its phenotypic contribution cannot be fully disentangled from the surrounding clonal background without functional validation.

Moreover, not all fluoroquinolone-resistant isolates carried the GyrA S83I mutation, consistent with previous reports (Lin et al., 2018). This observation indicates that fluoroquinolone resistance in *Elizabethkingia* is likely multifactorial. As clearly demonstrated in our updated resistome analysis (Supplementary Table 8), the universal presence of a massive array of intrinsic multidrug efflux pumps—particularly the broad-spectrum RND (e.g., *Mex* and *Ade* systems) and MFS (e.g., *emrB*) family transporters—undoubtedly plays a substantial role in expelling intracellular antibiotics. In addition to other QRDR mutations in GyrA, alterations in GyrB or topoisomerase IV, activation of efflux systems, regulatory changes, and potentially plasmid-mediated mechanisms may also contribute to resistance development (Acheampong et al., 2019; Chu et al., 2020; Liang et al., 2023; Zahari et al., 2023; Yang et al., 2025).

In summary, although *Elizabethkingia* accounts for a small proportion of clinical isolates, its capacity for clonal expansion and resistance evolution in healthcare settings warrants attention. Our findings indicate that fluoroquinolone resistance in hospital-associated *Elizabethkingia* results from the interplay between high-risk clonal dissemination and target-site mutation. Adaptive substitutions in *gyrA*, such as GyrA S83I detected in both *E. anophelis* and *E. meningoseptica*, reflect parallel evolution under antimicrobial selection pressure. Once selected, these mutations may be amplified through localized clonal expansion. Continued genomic surveillance is essential for identifying high-risk lineages and guiding antimicrobial and infection control strategies.

## Data Availability

The datasets presented in this study can be found in online repositories. The names of the repository/repositories and accession number(s) can be found below: https://www.ncbi.nlm.nih.gov/, PRJNA1312355.

## Footnotes

1
https://www.fda.gov/drugs/development-resources/tigecycline-injection-products?utm_source=chatgpt.com
