# Supplementary material for: Genomic characterization of respiratory Elizabethkingia: antimicrobial resistance and strain relatedness
Source: Front Microbiol. 2026 Apr 10;17:1815762. doi: 10.3389/fmicb.2026.1815762 (PMC13106410; doi:10.3389/fmicb.2026.1815762)
Supplement: Supplementary file 1 [file Data_Sheet_1.docx]

Supplementary Material

# Supplementary Tables

Supplementary Table S1 Genomic Characteristics of the 18 *Elizabethkingia* *s* Strains

| Strain | Genome Size (Mb) | G C content (%) | CDS | tRNA | rRNA | Completeness/Contamination (%) |
| --- | --- | --- | --- | --- | --- | --- |
| FK1 | 4.09 | 35.52 | 3754 | 46 | 3 | 100/0 |
| FK2 | 3.92 | 35.58 | 3619 | 43 | 3 | 100/0 |
| FK3_1 | 4.05 | 35.60 | 3752 | 42 | 3 | 100/0 |
| FK3_2 | 4.05 | 35.60 | 3756 | 44 | 4 | 100/0 |
| FK4 | 4.02 | 35.47 | 3710 | 43 | 3 | 100/1.08 |
| FK5 | 4.05 | 35.60 | 3734 | 41 | 3 | 100/0 |
| FK6_1 | 4.05 | 35.60 | 3734 | 41 | 3 | 100/0 |
| FK6_2 | 4.05 | 35.60 | 3757 | 42 | 4 | 100/0 |
| FK7 | 4.01 | 35.48 | 3701 | 43 | 3 | 100/1.08 |
| FK8 | 3.88 | 36.42 | 3503 | 43 | 5 | 100/0 |
| FK9 | 4.00 | 35.56 | 3684 | 42 | 4 | 100/0 |
| FK10 | 4.05 | 35.61 | 3737 | 44 | 3 | 100/0 |
| FK11 | 4.09 | 35.57 | 3788 | 42 | 3 | 100/0 |
| FK12 | 3.95 | 36.45 | 3557 | 43 | 5 | 100/0 |
| FK13 | 3.99 | 35.62 | 3660 | 43 | 3 | 100/1.08 |
| FK14 | 3.95 | 36.45 | 3552 | 43 | 3 | 100/0 |
| FK15 | 4.04 | 35.64 | 3735 | 46 | 3 | 100/0 |
| FK16 | 4.05 | 35.60 | 3742 | 43 | 3 | 100/0 |

# Supplementary Table S2. Pairwise core-genome SNP distances among *Elizabethkingia* clades

| Comparison | No. of pairs | Median  SNPs | IQR | Range  (min–max) |
| --- | --- | --- | --- | --- |
| Within_CladeB | 36 | 4 | 13 | 0–21 |
| CladeB_vs_CladeA | 18 | 23606 | 1020.5 | 23,077–24,148 |
| CladeB_vs_CladeC | 36 | 18666.5 | 1207.75 | 15,779–19,888 |
| CladeB_vs_CladeD | 27 | 206694 | 6230.5 | 200,907–207,202 |

Supplementary Table S3. Pairwise core-genome SNP distance matrix within Clade B

| Strain | FK10 | FK11 | FK16 | FK3_1 | FK3_2 | FK5 | FK6_1 | FK6_2 | FK9 |
| --- | --- | --- | --- | --- | --- | --- | --- | --- | --- |
| FK10 | 0 | 18 | 2 | 0 | 3 | 2 | 0 | 4 | 14 |
| FK11 | 18 | 0 | 19 | 18 | 20 | 19 | 18 | 21 | 12 |
| FK16 | 2 | 19 | 0 | 2 | 2 | 1 | 1 | 4 | 15 |
| FK3_1 | 0 | 18 | 2 | 0 | 3 | 3 | 0 | 4 | 14 |
| FK3_2 | 3 | 20 | 2 | 3 | 0 | 3 | 1 | 3 | 15 |
| FK5 | 2 | 19 | 1 | 3 | 3 | 0 | 0 | 4 | 14 |
| FK6_1 | 0 | 18 | 1 | 0 | 1 | 0 | 0 | 2 | 14 |
| FK6_2 | 4 | 21 | 4 | 4 | 3 | 4 | 2 | 0 | 16 |
| FK9 | 14 | 12 | 15 | 14 | 15 | 14 | 14 | 16 | 0 |

# Supplementary Table S4. Amino acid substitutions identified in GyrA among the 18 *Elizabethkingia* isolates included in this study

| Strain | GyrA Position | Nucleotide Change | Coding DNA Change | Protein Change | Species |
| --- | --- | --- | --- | --- | --- |
| FK1 | 841 | GCC->GTC | c.2522C>T | A841V | *E. anophelis* |
|  | 842 | ATT->GCT | c.2524_2525 | I842A | *E. anophelis* |
| FK2 | 841 | GCC->GTC | c.2522C>T | A841V | *E. anophelis* |
|  | 842 | ATT->GCT | c.2524_2525 | I842A | *E. anophelis* |
| FK3_1 | 83 | AGC->ATC | c.248G>T | S83I | *E. anophelis* |
| FK3_2 | 83 | AGC->ATC | c.248G>T | S83I | *E. anophelis* |
| FK4 | 841 | GCC->GTC | c.2522C>T | A841V | *E. anophelis* |
|  | 842 | ATT->GCT | c.2524_2525 | I842A | *E. anophelis* |
| FK5 | 83 | AGC->ATC | c.248G>T | S83I | *E. anophelis* |
| FK6_1 | 83 | AGC->ATC | c.248G>T | S83I | *E. anophelis* |
| FK6_2 | 83 | AGC->ATC | c.248G>T | S83I | *E. anophelis* |
| FK7 | 841 | GCC->GTC | c.2522C>T | A841V | *E. anophelis* |
|  | 842 | ATT->GCT | c.2524_2525 | I842A | *E. anophelis* |
| FK8 | 83 | AGC->ATC | c.248G>T | S83I | *E. meningoseptica* |
| FK9 | 83 | AGC->ATC | c.248G>T | S83I | *E. anophelis* |
| FK10 | 83 | AGC->ATC | c.248G>T | S83I | *E. anophelis* |
| FK11 | 83 | AGC->ATC | c.248G>T | S83I | *E. anophelis* |
| FK12 | 449 | ATG->GTG | c.1345A>G | M449V | *E. meningoseptica* |
| FK13 | 841 | GCC->GTC | c.2522C>T | A841V | *E. anophelis* |
|  | 842 | ATT->GCT | c.2524_2525 | I842A | *E. anophelis* |
| FK14 | 449 | ATG->GTG | c.1345A>G | M449V | *E. meningoseptica* |
| FK15 | 841 | GCC->GTC | c.2522C>T | A841V | *E. anophelis* |
|  | 842 | ATT->GCT | c.2524_2525 | I842A | *E. anophelis* |
| FK16 | 83 | AGC->ATC | c.248G>T | S83I | *E. anophelis* |

# Supplementary Table S5. Amino acid substitutions identified in ParC among the 18 *Elizabethkingia* isolates included in this study

| Strain | ParC Position | Nucleotide Change | Coding DNA Change | Protein Change | Species |
| --- | --- | --- | --- | --- | --- |
| FK1 | 196 | CCT->TCT | c.586C>T | P196S | *E. anophelis* |
|  | 375 | ACT->GCT | c.1123A>G | T375A |  |
| FK2 | / | / | / | / | *E. anophelis* |
| FK3_1 | 375 | ACT->GCT | c.1123A>G | T375A | *E. anophelis* |
|  | 429 | TCA->GCC | c.1285_1287 | S429A |  |
| FK3_2 | 375 | ACT->GCT | c.1123A>G | T375A | *E. anophelis* |
|  | 429 | TCA->GCC | c.1285_1287 | S429A |  |
| FK4 | / | / | / | / | *E. anophelis* |
| FK5 | 375 | ACT->GCT | c.1123A>G | T375A | *E. anophelis* |
|  | 429 | TCA->GCC | c.1285_1287 | S429A |  |
| FK6_1 | 375 | ACT->GCT | c.1123A>G | T375A | *E. anophelis* |
|  | 429 | TCA->GCC | c.1285_1287 | S429A |  |
| FK6_2 | 375 | ACT->GCT | c.1123A>G | T375A | *E. anophelis* |
|  | 429 | TCA->GCC | c.1285_1287 | S429A |  |
| FK7 | / | / | / | / | *E. anophelis* |
| FK8 | / | / |  | / | *E. meningoseptica* |
| FK9 | 375 | ACT->GCT | c.1123A>G | T375A | *E. anophelis* |
|  | 429 | TCA->GCC | c.1285_1287 | S429A |  |
| FK10 | 375 | ACT->GCT | c.1123A>G | T375A | *E. anophelis* |
|  | 429 | TCA->GCC | c.1285_1287 | S429A |  |
| FK11 | 375 | ACT->GCT | c.1123A>G | T375A | *E. anophelis* |
|  | 429 | TCA->GCC | c.1285_1287 | S429A |  |
| FK12 | / | / | / | / | *E. meningoseptica* |
| FK13 | 838 | ACC->ATC | c.2513C>T | T838I | *E. anophelis* |
| FK14 | / | / | / | / | *E. meningoseptica* |
| FK15 | 375 | ACT->GCT | c.1123A>G | T375A | *E. anophelis* |
| FK16 | 375 | ACT->GCT | c.1123A>G | T375A | *E. anophelis* |
|  | 429 | TCA->GCC | c.1285_1287 | S429A |  |

# Supplementary Table S6. Amino acid substitutions identified in ParE among the 18 *Elizabethkingia* isolates included in this study

| Strain | ParE Position | Nucleotide Change | Coding DNA Change | Protein Change | Species |
| --- | --- | --- | --- | --- | --- |
| FK1 | 128 | TTC->TGC | c.383T>G | F128C | *E. anophelis* |
| FK2 | / | / | / | / | *E. anophelis* |
| FK3_1 | / | / | / | / | *E. anophelis* |
| FK3_2 | / | / | / | / | *E. anophelis* |
| FK4 | / | / | / | / | *E. anophelis* |
| FK5 | / | / | / | / | *E. anophelis* |
| FK6_1 | / | / | / | / | *E. anophelis* |
| FK6_2 | / | / | / | / | *E. anophelis* |
| FK7 | / | / | / | / | *E. anophelis* |
| FK8 | / | / |  | / | *E. meningoseptica* |
| FK9 | / | / | / | / | *E. anophelis* |
| FK10 | / | / | / | / | *E. anophelis* |
| FK11 | / | / | / | / | *E. anophelis* |
| FK12 | 234 | AAA->AGA | c.701A>G | K234R | *E. meningoseptica* |
| FK13 | / | / | / | / | *E. anophelis* |
| FK14 | 234 | AAA->AGA | c.701A>G | K234R | *E. meningoseptica* |
| FK15 | / | / | / | / | *E. anophelis* |
| FK16 | / | / | / | / | *E. anophelis* |

Supplementary Table S4 Antimicrobial Susceptibilities of 18 *Elizabethkingia* Isolates Determined by the VITEK® 2 Compact System

| Antibiotics  /Strains | Piperacil  lin  /Tazobactam | Ceftazidime | Cefoperazone  /Sulbactam | Cefepime | Imipenem | Meropenem | Amikacin | Tobramycin | Ciprofloxacin | Levofloxacin | Doxycycline | Minocycline | Tigecycline | Colistin | Co-trimoxazole |
| --- | --- | --- | --- | --- | --- | --- | --- | --- | --- | --- | --- | --- | --- | --- | --- |
| FK1 | >=  128  R | >=  64  R | <=  8  S | >=  32R | >=  16  R | >=  16  R | >=  64  R | >=  16  R | <=  0.5  S | <=  4  S | <=  4  S | <=  2  S | <=  2  S | >=  16  R | <=  40  S |
| FK2 | >=  128  R | >=  64  R | <=  16  S | 16  I | >=  16  R | >=  16  R | >=  64  R | >=  16  R | 1  I | <=  4  S | / | / | / | >=  16  R | / |
| FK3-1 | >=  128  R | >=  64  R | >=  64  R | >=  32R | >=  16  R | >=  16  R | >=  64  R | >=  16  R | >=  4  R | >=  8  R | <=  4  S | <=  2  S | >=  8  R | >=  16  R | <=  40  S |
| FK3-2 | >=  128  R | >=  64  R | >=  64  R | >=  32R | >=  16  R | >=  16  R | >=  64  R | >=  16  R | >=  4  R | >=  8  R | 8  I | <=  2  S | <=  2  S | >=  16  R | >=  80  R |
| FK4 | >=  128  R | >=  64  R | <=  16  S | >=  32R | >=  16  R | >=  16  R | >=  64  R | >=  16  R | <=  0.5  S | <=  4  S | <=  4  S | <=  2  S | 4  I | >=  16  R | <=  40  S |
| FK5 | >=  128  R | >=  64  R | 32  I | 16  I | >=  16  R | >=  16  R | >=  64  R | >=  16  R | >=  4  R | >=  8  R | <=  4  S | <=  2  S | <=  2  S | >=  16  R | <=  40  S |
| FK6-1 | >=  128  R | >=  64  R | >=  64  R | >=  32R | >=  16  R | >=  16  R | >=  64  R | >=  16  R | >=  4  R | >=  8  R | <=  4  S | <=  2  S | >=  8  R | >=  16  R | <=  40  S |
| FK6-2 | >=  128  R | >=  64  R | >=  64  R | >=  32R | >=  16  R | >=  16  R | >=  64  R | >=  16  R | >=  4  R | >=  8  R | <=  4  S | <=  2  S | >=  8  R | >=  16  R | <=  40  S |
| FK7 | >=  128  R | >=  64  R | 32  I | >=  32R | >=  16  R | >=  16  R | >=  64  R | >=  16  R | <=  0.5  S | <=  4  S | 8  I | <=  2  S | 4  I | >=  16  R | >=  80  R |
| FK8 | >=  128  R | >=  64  R | 32  I | >=  32R | >=  16  R | 8  I | >=  64  R | >=  16  R | >=  4  R | >=  8  R | <=  4  S | <=  2  S | <=  2  S | >=  16  R | <=  40  S |
| FK9 | >=  128  R | >=  64  R | 32  I | >=  32R | >=  16  R | >=  16  R | >=  64  R | >=  16  R | >=  4  R | >=  8  R | <=  4  S | <=  2  S | 4  I | >=  16  R | <=  40  S |
| FK10 | >=128  R | >=  64  R | >=  64  R | >=  32R | >=  16  R | >=  16  R | >=  64  R | >=  16  R | >=  4  R | >=  8  R | <=  4  S | <=  2  S | 4  I | >=  16  R | <=  40  S |
| FK11 | >=  128  R | >=  64  R | >=  64  R | >=  32R | >=  16  R | >=  16  R | >=  64  R | >=  16  R | >=  4  R | >=  8  R | <=  4  S | <=  2  S | 4  I | >=  16  R | <=  40  S |
| FK12 | >=  128  R | >=  64  R | >=  64  R | >=  32R | >=  16  R | >=  16  R | >=  64  R | >=  16  R | >=  4  R | >=  8  R | <=  4  S | <=  2  S | >=  8  R | >=  16  R | <=  40  S |
| FK13 | >=  128  R | >=  64  R | >=  64  R | >=  32R | >=  16  R | >=  16  R | >=  64  R | >=  16  R | <=  0.5  S | <=  4  S | >=  16  R | <=  2  S | >=  8  R | >=  16  R | <=  40  S |
| FK14 | >=  128  R | >=  64  R | 32  I | >=  32R | >=  16  R | >=  16  R | >=  64  R | >=  16  R | >=  4  R | <=  4  S | >=  16  R | <=  2  S | >=  8  R | >=  16  R | >=  80  R |
| FK15 | >=  128  R | >=  64  R | 32  I | >=  32R | >=  16  R | >=  16  R | >=  64  R | >=  16  R | <=  0.5  S | <=  4  S | <=  4  S | <=  2  S | 4  I | >=  16  R | <=  40  S |
| FK16 | >=  128  R | >=  64  R | >=  64  R | >=  32R | >=  16  R | >=  16  R | >=  64  R | >=  16  R | >=  4  R | >=  8  R | >=  16  R | <=  2  S | >=  8  R | >=  16  R | >=  80  R |

S, susceptible; R, resistant; I, intermediate
